# Novel Brentuximab Vedotin Combination Therapies Show Promising Activity in Highly Refractory CD30+ Non-Hodgkin Lymphoma: A Case Series and Review of the Literature

**DOI:** 10.1155/2016/2596423

**Published:** 2016-10-11

**Authors:** Wilfred Delacruz, Robert Setlik, Arash Hassantoufighi, Shyam Daya, Susannah Cooper, Dale Selby, Alexander Brown

**Affiliations:** ^1^Hematology/Oncology Service, San Antonio Military Medical Center, Fort Sam Houston, San Antonio, TX 78234, USA; ^2^Department of Internal Medicine, San Antonio Military Medical Center, Fort Sam Houston, San Antonio, TX 78234, USA; ^3^Department of Pathology, San Antonio Military Medical Center, Fort Sam Houston, San Antonio, TX 78234, USA

## Abstract

Non-Hodgkin lymphomas (NHLs) are a heterogeneous group of hematologic malignancies which typically respond to standard first-line chemoimmunotherapy regimens. Unfortunately, patients with refractory NHL face a poor prognosis and represent an unmet need for improved therapeutics. We present two cases of refractory CD30+ NHL who responded to novel brentuximab vedotin- (BV-) based regimens. The first is a patient with stage IV anaplastic large cell lymphoma (ALCL) with cranial nerve involvement who failed front-line treatment with cyclophosphamide, doxorubicin, vincristine, etoposide, and prednisone (CHOEP) and second line cyclophosphamide, vincristine, doxorubicin, dexamethasone alternating with high-dose methotrexate (MTX), and cytarabine (hyperCVAD) with intrathecal- (IT-) MTX and IT-cytarabine, but responded when BV was substituted for vincristine (hyperCBAD). The second patient was a man with stage IV diffuse large B-cell lymphoma (DLBCL) with leptomeningeal involvement whose disease progressed during first-line rituximab, cyclophosphamide, doxorubicin, vincristine, and prednisone (R-CHOP) and progressed despite salvage therapy with rituximab, dexamethasone, cytarabine, and cisplatin (R-DHAP) in whom addition of BV to topotecan resulted in a significant response. This report describes the first successful salvage treatments of highly aggressive, double refractory CD30+ NHL using two unreported BV-based chemoimmunotherapy regimens. Both regimens appear effective and have manageable toxicities. Further clinical trials assessing novel BV combinations are warranted.

## 1. Introduction

Non-Hodgkin's Lymphomas (NHLs) are a heterogeneous group of hematologic neoplasms arising from lymphoid tissue. Aggressiveness of NHL subtypes and response to treatment are influenced by several prognostic factors including age, cell of origin, histopathology, stage, tumor proliferation rate, performance status, and associated genetic alterations [1]. At present, the response rates to first-line chemotherapy regimens are generally greater than 50% [2] and approximately 70% patients with aggressive NHLs will achieve a complete response [3]. In some cases, NHLs are refractory to standard first-line regimens. Unfortunately, these patients have a much lower chance for cure from salvage regimens. The 5-year prognosis of patients with primary refractory or relapsed aggressive NHL is dismal at roughly 10% [3]. Over the past several years, new therapies have been developed to overcome treatment resistance. These agents include next-generation anti-CD20 monoclonal antibodies (mAbs), antibody-drug conjugates, small-molecule inhibitors, radioimmunotherapy, and mAbs against non-CD20 extracellular markers.

Brentuximab vedotin (BV), or SGN-35, is a chimeric anti-CD30 mAb attached by a protease-cleavable linker to a microtubule disrupting agent, monomethyl auristatin E (MMAE) [4, 5]. BV binds to extracellular domain of CD30 and is internalized and subsequently transferred to the lysosome causing enzymatic cleavage of the linker peptide and release of MMAE into the cytosol. MMAE then binds to tubulin inhibiting microtubule polymerization and resulting in mitotic arrest and apoptosis in CD30-expressing lymphoma cells. MMAE is also diffusible across the cell membranes, which is thought to create a bystander antitumor effect into the tumor microenvironment [5]. Several studies show that BV has single agent activity in NHLs with high levels CD30 expression such as HL and ALCL as well as NHLs with very low CD30 expression or undetectable CD30 [6–9].

BV was approved by the Food and Drug Administration for three indications, (1) as single agent for Hodgkin's lymphoma (HL) after autologous stem cell transplant (ASCT) failure, (2) as a single agent for ALCL after multiagent chemotherapy failure, and (3) most recently as a consolidation therapy following ASCT in HL patients at risk of relapse or progression. In a pivotal Phase II study, BV was tested as single agent therapy in patients with relapsed or refractory Hodgkin's lymphoma (HL) after autologous stem cell transplantation (ASCT). This study showed significant efficacy with an overall response rate (ORR) of 75% and a complete remission (CR) rate of 34% [10]. A subsequent Phase III study (AETHERA) in HL patients at risk for relapse after ASCT showed a median progression-free survival (PFS) of 42.9 months when BV was used as consolidation therapy versus 24.1 months in the placebo group [11]. In a Phase II study, BV also showed significant activity in relapsed or refractory systemic anaplastic large cell lymphoma, with an ORR of 86% and a CR in 53% of patients [12]. In these studies, the most commonly reported side effect was peripheral neuropathy which affected 36–56% of patients treated with BV.

Several recent studies have shown promising activity using BV monotherapy in relapsed CD30+ NHL such as diffuse large B-cell lymphoma (DLBCL) and ALCL [5, 6, 13, 14]. There are also a few published studies evaluating the use of BV in combination with conventional cytotoxic chemotherapy. In a Phase 1 study, BV was combined with ABVD or AVD as up-front therapy for patients with newly diagnosed HL showing excellent efficacy with a CR of 94-95%, but with an increased pulmonary toxic effect in the BV + AVBD group [15]. Also, a Phase 3 trial comparing BV plus AVD to AVBD alone is currently underway [5, 16]. Further, a Phase 1 study assessing the safety and efficacy of BV given sequentially with standard CHOP or in combination with CHP (CHOP without vincristine) in patient with CD30+ mature T/NK-cell lymphoma showed an ORR of 100% (CR rate 88%) [17]. A Phase 3 study comparing BV plus CHOP to CHOP in the frontline treatment of mature T-cell lymphomas is also underway [18].

Herein we describe the successful salvage of two patients with aggressive, treatment refractory CD30+ NHL (ALCL and DLBCL) using novel combinations of BV-based chemotherapy. In both cases, BV was used “off-label” after exhausting standard treatment options. Both patients were informed that the use of BV would be “off-label” and extensively counseled prior to receiving the BV-based treatments described below. Written consent and verbal informed consent were obtained prior to initiating treatment. “Off-label” use of BV was done with the intent of the “practice of medicine” rather than for research purposes or the intention of changing BV approved indications or labeling. In accordance with good medical practice and published US Food and Drug Administration guidelines [19], no institutional review board approval or submission of an investigational new drug application was required.

## 2. Case Reports

**Figure 1:**
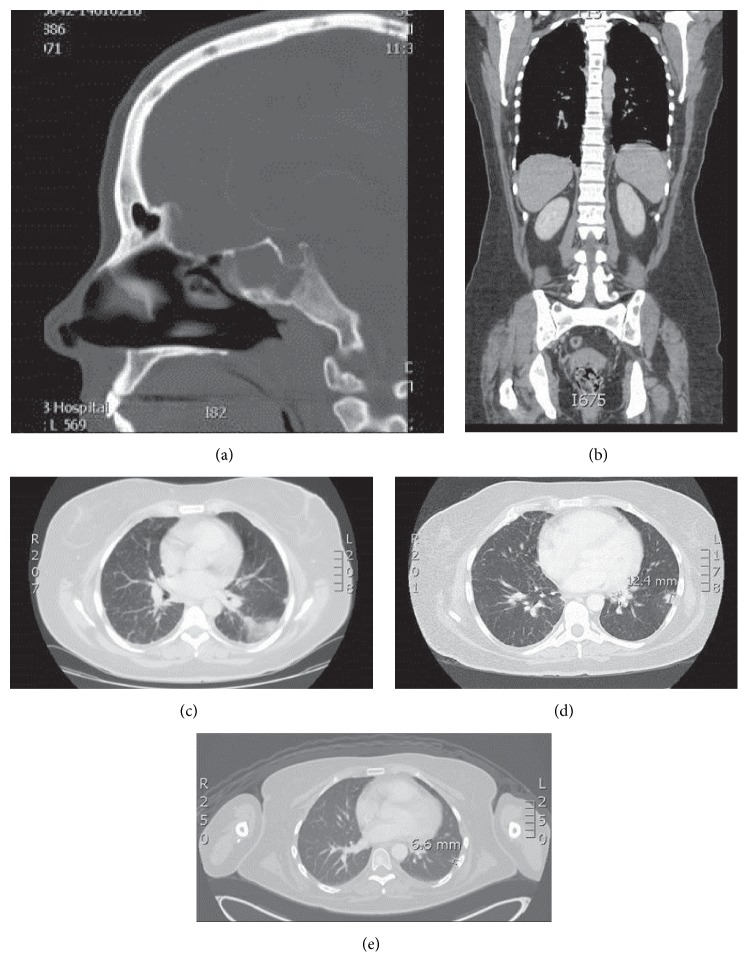
(a) Sagittal view of the sinus on computerized tomography (CT) showing the complete opacification of the right sphenoid sinus which was proven to be ALCL involvement by biopsy. (b) Axial view of CT C/A/P obtained at the time of diagnosis showing the diffuse osseous metastatic spread of disease involving vertebral bodies, pelvis, and humerus. (c) Representative lung field section of the CT C/A/P in (b) showing the numerous subcentimeter nodular densities within the lung parenchyma and along the margins of the fissures and pleural margins. Patient did not have significant lymphadenopathy (not shown). (d) Representative CT lung field performed after the first dose of hyperCVAD without BV showing interval progression of lung disease as well as development of new right hilar adenopathy (not shown). (e) Representative lung field of PET-CT (level approximates that of image (d)) showing resolution on the reticulonodular opacities and decrease in size of the largest subpleural nodule in the L lung. No PET avidity noted. Not shown are persistent diffuse lytic lesions that are not PET avid.

*Case  1.* The first case is of a 43-year-old previously female who initially presented with severe headaches and diplopia due to right abducens nerve palsy. On initial computerized tomography imaging (CT), she was found to have a large sphenoid mass, multiple pulmonary nodules, and multiple lytic bone lesions (Figure 1). During her subsequent assessment for tissue biopsy, she became severely hypoxic and was directly admitted to the intensive care unit (ICU) for hypoxic respiratory distress and required mechanical ventilation. Her ICU course was complicated by severe hyponatremia and sepsis secondary to* Pseudomonas *bacteremia successfully treated with cefepime and levofloxacin. A biopsy of the sphenoid sinus lesion was consistent with anaplastic large cell lymphoma (ALCL), with malignant cells positive for CD30+ and ALK-1+ immunohistochemical stains. She received her first cycle of cyclophosphamide, hydroxydaunorubicin, vincristine, etoposide, and prednisone (CHOEP) [20] while being intubated in the ICU. After 1 cycle of CHOEP, her condition stabilized and she was later discharged to follow up at our institution for second opinion and evaluation for autologous stem cell transplantation. Despite the well-described chemosensitivity of ALK-positive ALCL and the relatively favorable prognosis of this entity compared to ALK negative ALCL, our patient presented with fulminant, multisystem disease. Due to the aggressiveness of her disease coupled with the possibility of central nervous system (CNS) involvement, our team decided to escalate her regimen to include CNS-penetrating agents using standard hyperCVAD with high dose methotrexate (MTX) and cytarabine with intrathecal (IT) MTX and cytarabine [21]. The cycle of CHOEP she received in the ICU was counted as cycle 1 of hyperCVAD. On her follow-up after cycle 2, she presented with a clinically palpable lymph node in her scalp; she had a gradual increase of plasma lactate dehydrogenase (LDH) from normal levels peaking to 347 (U/L, range 135–225) several days after completion of hyperCVAD. CT showed confirmed progression of her pulmonary disease (Figure 1). Since her disease was strongly CD30-positive and her anticipated prognosis was extremely poor given demonstrated refractoriness of disease to very intensive chemotherapy regimen, we decided to add BV to the hyperCVAD regimen. Due to the overlapping neurotoxicity between BV and vincristine, vincristine was removed from her hyperCVAD regimen (herein referred to as “hyperCBAD”).

The hyperCBAD regimen is modified hyperCVAD with two regimens alternating on a 21-day cycle. The first regimen (A) consists of cyclophosphamide 300 mg/m^2^ intravenous given every 12 hours on days 1 to 3, MESNA 600 mg/m^2^/day continuous infusion given on days 1 to 3 six hours after cyclophosphamide, doxorubicin 50 mg/m^2^ intravenous given on day 4, dexamethasone 40 mg given on days 1 to 4 and days 11 to 14, IT-MTX 12 mg given on day 2, and IT-cytarabine 100 mg given on day 8. BV 1.8 mg/kg was given on day 1 one hour after cyclophosphamide. G-CSF support with pegfilgrastim 6 mg was given subcutaneously on day 5. The second regimen (B) consists of high dose MTX (200 mg/m^2^ given over 2 hours on day 1 followed by 800 mg/m^2^ over 22 hours), cytarabine 3000 mg/m^2^ given intravenously every 12 hours on days 2 and 3, methylprednisolone 50 mg given intravenously twice daily on days 1 to 3, and IT-MTX 12 mg given on day 2. Leucovorin 50 mg intravenously was given 12 hours from the start of MTX infusion followed by 50 mg every 6 hours until serum MTX is less than 0.1. BV 1.8 mg/kg was given intravenously on day 1. G-CSF support with pegfilgrastim 6 mg was given on day 4. For both regimens, the patient was given prophylactic antimicrobials with levofloxacin 500 mg daily, posaconazole 300 mg tab daily, acyclovir 400 mg twice daily, and atovaquone 750 mg daily.

The PET/CT after 3 cycles of alternating regimens A and B of hyperCBAD showed no metabolic evidence of lymphoma. The widespread osseous lytic lesions persisted but did not show FDG-PET avidity. The sphenoid sinus mass also persisted but no FDG avidity was demonstrated. The right abducens nerve palsy resolved. She then received consolidation radiation therapy to right sphenoid sinus. Endoscopic evaluation of the sphenoid sinus was unremarkable. Given concern that high dose chemotherapy and autologous stem cell transplant would result in excessive toxicity having just completed the hyperCBAD regimen and considering that the patient achieved a complete remission, our team decided to hold autologous stem cell transplantation. Instead, she was started on single agent BV maintenance (1.8 mg/kg every 21 days). After 5 cycles of maintenance BV, she opted to stop treatment due to worsening peripheral neuropathy. After two months of observation, she relapsed in the right temporal parietal lobe presenting with severe headaches and neurologic deficits. She was treated with 2 cycles of high dose MTX (8,000 mg/m^2^ every 2 weeks) which stabilized the CNS disease. She was then treated with salvage chemotherapy with cytarabine (3 gm/m^2^ given on days 1 and 2), thiotepa (40 mg/m^2^ given on day 2), IT liposomal cytarabine 50 mg on day 3, and dexamethasone 4 mg orally given twice daily on days 3 to 7 [22]. Unfortunately, the patient's performance status continued to decline and she opted for best supportive care with home hospice and died at home.

**Figure 2:**
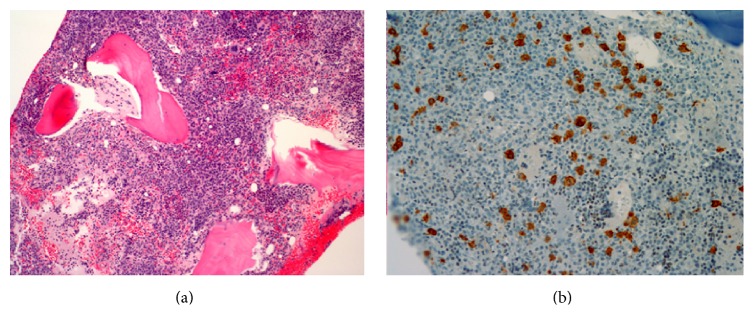
(a) Hypercellular bone marrow core biopsy demonstrating interstitial proliferation of large lymphoma cells. Hematoxylin and eosin stained bone marrow core biopsy (×100). (b) CD30 immunohistochemical stain of the bone marrow core biopsy. A subset of the large lymphoma cells are CD30 positive. Ber-H2 antibody (×200).

*Case  2.* The second patient is a 60-year-old male with history of coronary artery disease, hypertension, and hyperlipidemia, who was referred to our clinic by his cardiologist for evaluation of diffuse lymphadenopathy. One week prior to presenting to his cardiologist, he reported having acute viral illness, consisting of acute diarrhea and nausea. His symptoms improved after four to five days, but then he developed new cervical and axillary lymphadenopathy, difficulty swallowing, decreased appetite, and an eight-pound weight loss over two weeks. Initial workup was negative for Epstein Bar Virus (EBV), cytomegalovirus (CMV), and Human Immunodeficiency Virus (HIV) infection. Lactate dehydrogenase and uric acid levels were elevated. Review of his peripheral blood smear was notable for large, atypical lymphocytes. Subsequent flow cytometry analysis of the peripheral blood was significant for a CD5+ monoclonal B-cell population with lambda light chain restriction. CT of his chest, abdomen, and pelvis showed diffuse lymphadenopathy (Figure 2(a)). The bone marrow core biopsy showed a hypercellular marrow (95%) (Figure 2): 50% of the cells were large lymphoid cells positive for CD5, CD20, and dimly BCL 6 positive. These same cells were also found in the cerebrospinal fluid (CSF) analysis.

He was diagnosed with stage IV nongerminal center DLBCL with leptomeningeal involvement and was started on standard rituximab, cyclophosphamide, Adriamycin, vincristine, and prednisone (R-CHOP) with IT-MTX [23]. His first cycle of R-CHOP was complicated by pancytopenia and neutropenic fever which was successfully treated with broad spectrum antibiotics. Upon completion of his second IT-MTX treatment, no monoclonal B-cells were seen in his CSF by flow cytometry and cytology. After 3 cycles of R-CHOP and five doses of IT-MTX, the patient subsequently developed a right cranial nerve (CN) III palsy and right eyelid lag despite significant response in the lymphadenopathy. Given clinical evidence of leptomeningeal disease, we decided to continue R-CHOP but added high dose intravenous MTX every three weeks, given one week after R-CHOP. High dose MTX was added because of known central nervous system (CNS) penetration and activity. We also added IT-cytarabine along with IT-MTX alternating between cycles.

**Figure 3:**
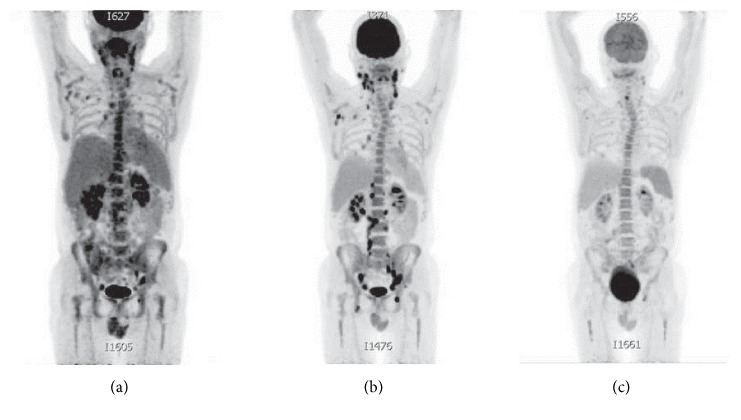
Staging NM-PET imaging after chemotherapy. (a) PET staging after 2 cycles of R-CHOP: partial anatomic response to therapy. Interval decrease in diffuse lymphadenopathy in comparison to initial CT. (b) Staging PET-CT after 5 cycles of R-CHOP, 1 cycle of R-DHAP, and IT methotrexate and cytarabine treatments. Widespread diffuse adenopathy with overall progression. (c) Staging after 2 cycles of brentuximab and topotecan. Significant decreases in size and metabolic activity of the diffuse adenopathy were observed.

Despite receiving two more doses of R-CHOP, two doses of IT-MTX and Ara-C, and three doses of high dose intravenous MTX, he developed progressive lymphadenopathy and additional neurologic deficits (right CN VI palsy and right arm weakness in addition to his right CN III palsy and right eye lid lag). Restaging PET/CT confirmed progression of his diffuse lymphadenopathy with no malignant foci in the brain or spine. Despite progression of disease, his performance status remained intact with an Eastern Cooperative Oncology Group (ECOG) performance status of 1. The patient was then treated with salvage chemotherapy using R-DHAP (rituximab, dexamethasone, high dose Ara-C, and cisplatin) [24] and IT-depot liposomal cytarabine [25]. He developed acute kidney injury, likely from cisplatin, and new hoarseness due to involvement of the recurrent laryngeal nerve. Further treatment with R-DHAP salvage chemotherapy was stopped due to continued, progressive disease (Figure 3(b)). Despite disease progression on cisplatin-based salvage chemotherapy, the patient's performance status was still excellent and the patient expressed willingness to pursue alternative therapy. Reanalysis of the bone marrow specimen revealed that approximately 10–15% of the lymphoma cells expressed CD30 by immunohistochemistry (Figure 2). We then decided to combine BV (1.8 mg/kg given on day 1) with topotecan (1 mg/m^2^ given on days 1–6) on a 21-day schedule. Topotecan was chosen for its ability to penetrate the CNS [26] and the dose and schedule were adopted from the TTR (taxol, topotecan, and rituximab) regimen [27]. Upon completion of cycle 1 of this regimen, he developed Grade 2 vertigo. A magnetic resonance imaging scan (MRI) of his brain did not show a CNS lesion. The patient otherwise continued to have good functional status and treatment was continued. A staging PET/CT performed after completion of the 2nd cycle of TTR showed significant improvement in adenopathy (Figure 3(c)), but he developed Grade 2 diarrhea, Grade 3 neutropenia, Grade 3 anemia, Grade 2 thrombocytopenia, and Grade 2 vertigo, with a subsequent decrease in his ECOG performance status from 1 to 2. He otherwise had no new neurological deficits. Repeated CSF analysis showed no malignant lymphocytes. He received the third dose of BV and topotecan and was evaluated for stem cell harvest for possible ASCT. Unfortunately, the patient developed episodic syncope, lightheadedness, and diaphoresis, thought to be due to autonomic dysfunction, and his performance status declined drastically. Although brain and spinal MRI did not reveal any focal abnormalities and CSF analysis was negative, clinical progression of the leptomeningeal disease could not be ruled out. He was empirically treated for adrenal insufficiency with a trial of fludrocortisone which was ineffective. At this point, he declined further treatment and was transitioned to best supportive care with home hospice. Two months after discontinuing therapy, he died at home.

## 3. Discussion

Primary refractory and relapsed aggressive NHLs are challenging to treat and patients with these disorders face a grim prognosis. Upon relapse or when the disease is refractory to standard therapy, high dose chemotherapy with autologous stem cell rescue may offer the best chance for cure in medically fit patients [28]. However, obtaining an adequate response prior to ASCT is often very difficult and even when a response is achieved, outcomes are suboptimal. For example, patients with DLBCL who relapse after rituximab containing frontline therapy such as R-CHOP face approximately an 80% chance of relapse despite salvage chemotherapy and high dose chemotherapy with autologous stem cell transplant [24]. Thus, novel salvage therapies for relapsed-refractory NHL are desperately needed.

BV has been used as a single agent for relapsed ALCL and DLBCL. It is also currently being investigated in the frontline setting for the treatment of Hodgkin's lymphoma (ECHELON-1 [16]) and CD30+ peripheral T-cell lymphoma (ECHELON-2 [18]) and as salvage therapy for relapsed CD30+ DLBCL where ifosfamide, carboplatin, and etoposide (ICE) are given following BV if disease persists after 2 cycles of BV (Clinical trial no. NCT01508312) [29]. A report by Heidegger et al. also showed that BV combined with DHAP used in a patient with primary refractory ALCL was effective, allowing for consolidation with high dose chemotherapy and ASCT without significant toxicities [30]. Given previous reports of BV combined with cytotoxic chemotherapy showing manageable toxicities and promising benefit, there is an opportunity to combine BV with other active chemotherapy agents in CD30+ diseases when standard treatments have been exhausted. In the two cases described above, we combined BV in unique ways with significant resultant clinical activity, in one case of ALCL using “hyperCBAD” and in one case of DLBCL combining BV with topotecan. Such responses could serve as a bridge to high dose chemotherapy with autologous stem cell transplant or to allogeneic stem cell transplant. Unfortunately, our patients both succumbed to CNS disease. In each case, however, extracranial disease was otherwise well controlled by combining BV with active agents.

We did not see an appreciable increase in intolerable toxicities in either patient when BV was used in combination with other chemotherapeutics. The patient who received “hyperCBAD” experienced peripheral neuropathy typically seen in patients receiving BV. The neuropathy progressively worsened while receiving maintenance BV resulting in discontinuation of treatment. The patient also had anemia and thrombocytopenia requiring transfusions and febrile neutropenia, requiring empiric antibiotic treatment. These toxicities are not uncommon toxicities seen in patients receiving an intensive regimen such as hyperCVAD. The patient who received the combination of BV and topotecan did not develop neuropathy. He developed hematologic toxicities including anemia, thrombocytopenia, and neutropenia which are common in patients receiving topotecan. He also experienced diarrhea which was easily managed with an antimotility agent. It is unclear whether the vertigo was related to the leptomeningeal disease, the multiple intrathecal chemotherapy treatments he received, or the combination of BV and topotecan.

The relationship between the degree of CD30 positivity and the response to BV is unclear. The mechanism by which BV works in NHL with low expression of CD30 or NHL not expressing CD30 using standard assays is yet to be determined. In our patient with DLBCL, CD30 IHC testing was not initially done as CD30 is not part of the standard IHC tests for DLBCL in our facility. This case also underscores the need to include CD30 as part of the standard IHC testing in NHLs.

In conclusion, these cases as well as burgeoning ongoing research demonstrates that, combining BV with active, intensive chemotherapy regimens can be used in primary refractory CD30+ NHL with improved efficacy compared with traditional treatment and with acceptable and manageable toxicities. Patients with relapsed-refractory NHL unfortunately face a poor prognosis and further investigation is clearly needed to fully evaluate the safety and efficacy of BV combination regimens.
